# Education and the island of misfit toys

**DOI:** 10.1007/s40037-016-0309-x

**Published:** 2016-10-20

**Authors:** Steven J. Durning, Holly S. Meyer, Pim W. Teunissen

**Affiliations:** 1 Department of Medicine, Uniformed Services University of the Health Sciences, Bethesda, MD USA; 2 School of Health Professions Education, Maastricht University, Maastricht, The Netherlands

Teaching is a core activity for health professionals that pervades our interactions with patients, learners and colleagues and is explicitly outlined as a core competency under the CanMEDS framework. Unfortunately, as Kloek and colleagues point out in this issue of Perspectives on Medical Education [1], teaching is typically assumed to be a skill present in medical graduates and limited education is invested in it. The authors [1] state that ‘training in the medical workforce is not paralleled by the training of their educators in the skills of teaching.’ This is somewhat understandable given how many years are invested in becoming a health professional and pressures on institutions to generate clinical revenues and win grant funding.

As a consequence, at many schools (at least in the US) teaching is often assumed to be achievable with any health professional with content expertise (e. g. understanding of medicine). Scholars have studied pedagogical content knowledge – a model aligning educators’ subject matter knowledge and pedagogical knowledge [2]. The pedagogical content knowledge model has effectively been used in educational settings to reframe individuals’ preconceptions, based on their own experiences about how material should be taught [3].

Indeed, up until relatively recently, being a medical or other health professional educator as a career has been seen as being akin to being on the Island of Misfit Toys. As portrayed in a movie, this is an island where toys with defects live, with the hope of someday being chosen by Santa for a child on Christmas day. Thankfully, this belief has changed and the authors add another piece of evidence [1] supporting this change in describing their innovative approach and outcomes.

According to the authors [1], there are now over 30 medical education journals and an increasing number of Master and PhD programmes in health professions education. In this commentary, we will build on the findings outlined by the authors [1] expanding the argument of why teaching matters, particularly in our contemporary health professional education and practice settings. We will conclude with an argument for why education can improve patient care and potential next steps for research.

## Short- to intermediate-term benefits

Prior work has demonstrated the benefits of teaching by (near) peers on learning; this impact likely benefits the learning of not only the students but also the teachers themselves [4, 5]. Thus, there appear to be growing empirical as well as theoretical benefits to both the teacher and the learners [6]. Additional benefits of teaching include exposing learners to careers in health professions education which is needed as demonstrated, for example, by the shortages of physicians and nurses in the US. Teachers also serve as role models for learners.

There is also the benefit of promoting teaching skills as well as needed scholarship on this topic through innovative coursework such as outlined by the authors [1]. This scholarship has the potential of facilitating cross-discipline and cross-institution collaboration, particularly through the emergence of innovative technology and health professional degree programmes [1]. The authors also add to the literature by providing data to suggest that taking part in a teaching rotation may lead to enhanced interest and enthusiasm in teaching, which can help meet needed workforce requirements.

## Longer-term benefits

The authors provide a longer-term outcome of teaching – more likely to remain active as teachers. Given that sufficient teachers are critical to meeting workforce needs, this is an important finding for society. Additionally, such programmes have the potential to impact policy decisions such as how to address shortages in physicians in the United States.

Another potential benefit may be that those who understand education may become better learners. In an age where life-long learning is required of all health professionals, having the skills to keep up to date, to learn from your work and to apply that directly to your future work and professional development is of great benefit. This would be an area for future investigation.

Additionally, through educational programme rotations such as the authors have undertaken as well as other training programmes (e. g. PhD in health professions education), future health professionals (e. g. physicians) learn about the importance of constructive learning environments. The sociocultural, historical, and complex learning theories that are gaining ground in health professions education help teachers to understand what constructive learning environments are made of and arguably an open, constructive learning environment is also an environment that is conducive to safe and continuously improving patient care. These indirect effects are, however, much harder to ‘prove’ fellowships) can impact patient care. This is a more difficult proposition to support empirically but it is critical for meeting societal needs. The steps in this argument are: health professional students are bright and motivated, these students will learn more from being taught by trained educators and finally, better trained learners will take better care of patients.

Several studies suggest that health professionals are bright and motivated. Stringent selection exists for medicine and dentistry, for example, with graduation rates of over 90 % at typical institutions in the US. The programmes are rigorous and the amount of time dedicated to becoming a health professional spans many years. As the overwhelming majority of students who enter medical or dental school become licensed health professionals, we believe the evidence for this step is fairly strong.

In terms of learning more by being taught by trained educators (e. g. those with Master or PhD in health professions education degrees or even following a course such as the authors suggest), there is literature from the field of faculty development to suggest that being taught by educationally trained faculty (individuals with pedagogical content knowledge) results in improved learning. Also, without training, individuals attempting to teach have considerable biases regarding the teaching strategies employed based on their own learning experience [3]. Furthermore, the fact that educational programmes, such as health professions education degrees, are growing globally suggests that we endorse this idea on an international scale.

The argument that learners who are taught by individuals and/or have advanced education training themselves take better care of patients is worthy of additional exploration. Work by Holmboe and Kogan provides some preliminary evidence to suggest that this may be so by demonstrating that faculty who performed better on an OSCE provided better feedback (e. g., teaching) to residents [7]. It makes sense that educational skills correlate with clinical skills. But does advanced education training lead to more direct benefits to care, for example, through improved communication with patients about their disease and treatment? Studies are clearly needed although it makes intuitive sense that health professionals with additional training (e. g. advanced degrees) in education would be more effective at teaching them about their diagnosis, shared decision making, and helping patients to navigate our complex systems of care. These hypotheses should be explored with further investigation.
